# Well Put Together—A Guide to Accessorizing with the Herpesvirus gH/gL Complexes

**DOI:** 10.3390/v14020296

**Published:** 2022-01-30

**Authors:** Gonzalo L. Gonzalez-Del Pino, Ekaterina E. Heldwein

**Affiliations:** Department of Molecular Biology and Microbiology, Tufts University School of Medicine, Boston, MA 02111, USA; gonzalo.gonzalez_del_pino@tufts.edu

**Keywords:** viral entry, glycoproteins, herpesviruses, gH/gL, membrane fusion

## Abstract

Herpesviruses are enveloped, double-stranded DNA viruses that infect a variety of hosts across the animal kingdom. Nine of these establish lifelong infections in humans, for which there are no cures and few vaccine or treatment options. Like all enveloped viruses, herpesviruses enter cells by fusing their lipid envelopes with a host cell membrane. Uniquely, herpesviruses distribute the functions of receptor engagement and membrane fusion across a diverse cast of glycoproteins. Two glycoprotein complexes are conserved throughout the three herpesvirus subfamilies: the trimeric gB that functions as a membrane fusogen and the heterodimeric gH/gL, the role of which is less clearly defined. Here, we highlight the conserved and divergent functions of gH/gL across the three subfamilies of human herpesviruses by comparing its interactions with a broad range of accessory viral proteins, host cell receptors, and neutralizing or inhibitory antibodies. We propose that the intrinsic structural plasticity of gH/gL enables it to function as a signal integration machine that can accept diverse regulatory inputs and convert them into a “trigger” signal that activates the fusogenic ability of gB.

## 1. Introduction

Herpesviruses are double-stranded DNA, enveloped viruses that infect many species causing life-long infections. Nine herpesviruses from three subfamilies, namely alphaherpesviruses/*alphaherpesvirinae*, betaherpesviruses/*betaherpesvirinae*, and gammaherpesviruses/*gammaherpesvirinae*, infect humans. These viruses present a serious health challenge globally [1]. Alphaherpesviruses herpes simplex viruses 1 and 2 (HSV-1 and HSV-2) and varicella-zoster virus (VZV) can cause not only acute mucocutaneous lesions but also long-term neurological damage due to encephalitis [2,3] and shingles [4]. The betaherpesvirus human cytomegalovirus (HCMV) causes diseases in immunocompromised patients [5] and is the leading cause of birth defects due to congenital infection [6]. The gammaherpesviruses Epstein–Barr virus (EBV) and Kaposi’s sarcoma-associated herpesvirus (KSHV) cause lymphomas and epithelial malignancies [7,8]. Altogether, roughly 90% of the human population is permanently infected with at least one herpesvirus [9].

Like other enveloped viruses, herpesviruses enter cells by fusing their lipid envelopes with a host cell membrane, either the plasma membrane or the membrane of an endocytic vesicle/endosome after internalization [10,11], which releases the viral capsid and associated proteins into the host cytoplasm. Entry of enveloped viruses into a target cell proceeds through a sequence of events that begins with the binding of a viral receptor-binding glycoprotein to a cognate host cell receptor and culminates with membrane fusion by a viral fusogen, a glycoprotein that undergoes large conformational changes to bring apposing membranes into proximity until they fuse. In most enveloped viruses, such as influenza [12,13], human immunodeficiency virus (HIV) [14,15,16], and vesicular stomatitis virus (VSV) [17,18], the receptor-binding and fusogenic functions are located within the same viral glycoprotein, albeit sometimes in distinct subunits. However, in herpesviruses these functions are distributed among several glycoproteins, some conserved and others divergent, which could account for the broad tropism of the *Herpesviridae* family.

The minimal core entry machinery of the *Herpesviridae* is composed of three conserved proteins, gB, gH, and gL [19]. gB is a ~300-kiloDalton (kDa) homotrimer composed of a large ectodomain, a single-spanning transmembrane helix, and a cytoplasmic domain. gB functions as a viral fusogen—a molecular machine that mediates membrane fusion by refolding from the metastable prefusion form to the stable postfusion form. Structural analyses of many fusogens from all three subfamilies have permitted reconstruction of the steps in the fusion process [10]. Fusogens are kept in a compact, metastable, “primed” state until switched on either by an increase in environmental acidity [15,20,21] or by binding to a target cell receptor [22,23]. Once activated, fusogens undergo conformational changes to expose their secluded hydrophobic fusion segments (peptides or loops) and insert them into the target membrane. This coincides with the formation of an extended, high-energy intermediate. Collapse of this intermediate into a stable, hairpin-like postfusion conformation is thought to provide sufficient free energy to bring the opposing membranes into proximity so that they merge. gB is a class III fusogen, along with the VSV G and baculovirus gp64 [24,25]. Unlike members of classes I or II, class III fusogens do not require priming by the cleavage of the fusogen itself (class I) or its associated chaperones (class II) [10]. Moreover, unlike most fusogens, gB is not activated by an increase in acidity or by binding to a target cell receptor or a co-receptor. Instead, gB is thought to be activated by the heterodimeric complex composed of two viral glycoproteins, gH and gL, conserved across all *Herpesviridae* [19].

**Figure 1:**
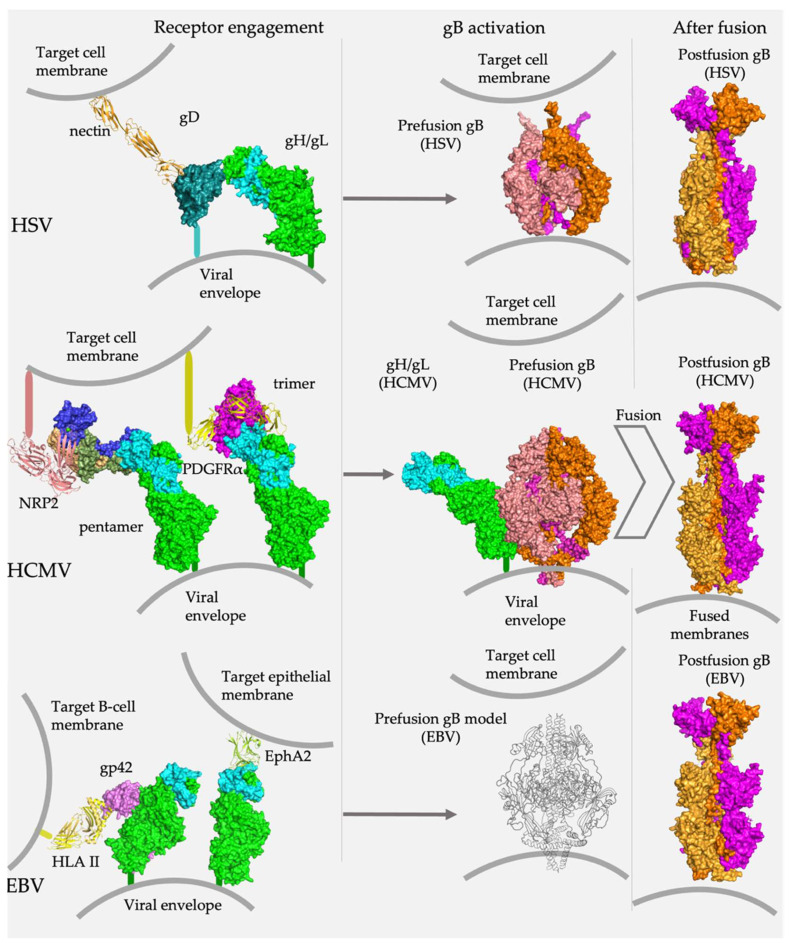
gH/gL is a central player in herpesvirus entry. Across all subfamilies, gH/gL connects binding of host cell receptors with membrane fusion. In alpha- and betaherpesviruses, gH/gL binds receptor-binding accessory proteins, HSV gD and HCMV gO and UL128/UL130/UL131. In gammaherpesviruses, gH/gL either binds a receptor-binding accessory protein (EBV gp42) or directly engages the host cell receptor (EphA2). In all cases, gH/gL interacts with gB, presumably, relaying the “trigger” signal that leads to the conformational rearrangements that effect membrane fusion. Research Collaboratory for Structural Bioinformatics Protein Data Bank Identifiers (RCSB PDB IDs) [27]: gD/nectin (4MYW) [28], HSV-2 gH/gL (3M1C) [29], HCMV gH/gL/gO (7LBE) [30], HCMV gH/gL/UL128/UL130/UL31 (5VOB) [31], EBV gH/gL/EphA2 (7CZE) [32], prefusion HSV gB (6Z9M) [33], postfusion HSV gB (2GUM) [24], prefusion HCMV gB (7KDP) [34], postfusion HCMV gB (5CXF) [35], postfusion EBV gB (3FVC) [36]. The structure of HCMV gH/gL was extracted from the HCMV gH/gL/UL128/130/131 complex (5VOD). The structure of EBV gH/gL/gp42/HLA II was assembled from gH/gL/gp42/E1D1 (5T1D) and gp42/HLA II (1KG0). The schematic of prefusion EBV gB was modeled on the HCMV prefusion gB structure.

gH is a ~90-kDa glycoprotein with a large ectodomain, a single-spanning transmembrane helix, and a short cytoplasmic tail. Correct folding, trafficking to the cell surface, and function require that gH bind to gL, a soluble, ~25-kDa glycoprotein. The gH/gL heterodimer is at the center of herpesvirus entry because it interacts with several key participants in the fusogenic cascade (Figure 1). On the one hand, it binds the “upstream” participants, either host-cell receptors or the viral receptor-binding accessory proteins, depending on the specific herpesvirus and host cell type. On the other hand, it also binds the fusogen gB, located “downstream” and, presumably, triggers conformational rearrangements that bring about fusion. How the host-cell binding signal is transmitted to the fusogen remains unclear [26].

In this review, we compare the conserved and divergent properties of the gH/gL complexes across the *Herpesviridae* subfamilies. We focus on the structural and biochemical analyses of the gH/gL complexes and their interactions with the viral receptor-binding accessory proteins and host cell receptors. We also analyze their interactions with antibodies and highlight mechanistic insights afforded by these studies. We propose that gH/gL is an unusual viral machine that has intrinsic structural plasticity that enables it to accept a broad range of regulatory inputs and convert them into a “trigger” signal interpretable by the conserved herpesvirus fusion machine, gB.

## 2. The gH/gL Structure

**Figure 2:**
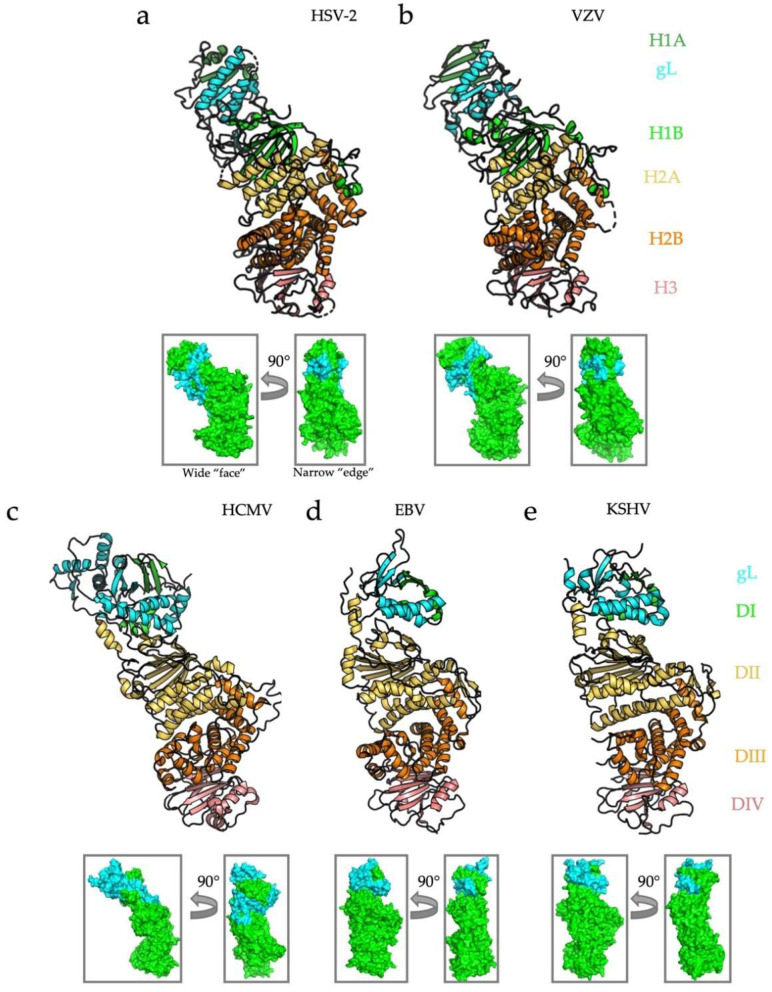
Structures and domain architecture for known human herpesvirus gH/gL heterodimers. Structures of five gH/gL heterodimers from human herpesviruses HSV-2, VZV, HCMV, EBV, and KSHV. In cases where structures were determined in complex with a receptor or an accessory protein(s), only gH and gL are shown, for clarity. Although sequences are not well conserved across *Herpesviridae*, the structures share remarkable similarities. The domains of gH adopt a linear arrangement, with domain H1 (DI and the N terminus of DII in beta and gamma-herpesviruses) co-folding with gL and making up the “N-terminal module.” HSV-2 (**a**), VZV (**b**), and HCMV (**c**) gH/gL are “kinked” whereas EBV (**d**) and KSHV (**e**) are cylindrical. All structures are aligned in PyMol [40]. The small insets indicate the two main functional sides of the complex: the wide “face” and the narrow “edge.” RCBS PDB IDs: HSV-2 (3M1C) [24], VZV (4XHJ) [37], HCMV (5VOB) [38], EBV (3PHF) [39], and KSHV (7CZF) [32].

Structures of the gH/gL complexes have been determined for the HSV-2 [29], VZV [37], HCMV [38], EBV [39], and KSHV [32] homologs (Figure 2). In all instances, the heterodimer adopts an elongated structure, in which the gH N terminus and gL are located distal from the membrane. In the alphaherpesvirus HSV-2, gH is divided into three distinct domains: the N-terminal, β-sheet-rich domain H1 that sandwiches gL between its two lobes, H1a and H1b; the globular domain H2 composed of 13 alpha helices and subdivided into H2a, a four-helix bundle, and a crescent-shaped H2b; and domain H3, a 10-strand β-sandwich (Figure 2a) [29]. gL co-folds with domain H1, forming a five-strand β-sheet spanning both gH and gL. The gH/gL from another alphaherpesvirus, VZV, aligns nearly perfectly with HSV-2 gH/gL, with the major difference being a larger β-sheet in H1 [37] (Figure 2b). In both alphaherpesviruses, gH/gL is kinked at the juncture between domains H1 and H2, giving it a boot-like appearance.

In beta- and gammaherpesviruses, gH/gL is, instead, divided into four domains that nonetheless closely resemble their alphaherpesviral counterparts. In HCMV, a large gL also forms a five-strand β-sheet with the fifty N-terminal residues of the gH domain DI (Figure 2c). The C-terminal α-helices of gL interact extensively with the top of the gH domain DII, similarly to the HSV homologs. DII is made up of two distinct regions–a seven-strand N-terminal β-sheet positioned above a three-helix bundle. For reference, the N-terminal β-sheet and the helical bundle in HCMV gH domain DII corresponds to subdomains H1b and H2a in HSV gH, respectively [26]. The helical bundle of domain DII packs against a large helical domain, DIII (H2b in HSV gH). Domain DIV in HCMV gH is also a ten-strand β-sandwich that corresponds to the domain H3 in HSV gH. The entire structure is also kinked at the junction of the β-sheet and the helical bundle in domain DII, but to a lesser extent than in HSV and VZV gH/gL.

EBV and KSHV gH/gL have a much smaller DI-gL module than their alpha- and betaherpesvirus counterparts (Figure 2d,e). In both cases, gH DI and gL still co-fold into a β-sheet that rests on a helical platform made up by gL and gH DI. gH domain DII in gammaherpesviruses retains the β-sheet/helical bundle configuration. DIII is the large helical domain, and DIV is a membrane-proximal β-sandwich. In contrast to HSV, VZV, and HCMV, however, EBV and KSHV gH/gL stand fully upright with hardly any kink in DII. In all cases, the gH/gL structure resembles a rectangular prism oriented upright on the virion envelope, with two wide and two narrow edges along the long, vertical axis (Figure 2, small insets). As we discuss later in this review, these sides correspond to distinct functional regions on the heterodimer.

## 3. gH/gL Interactions with Receptor-Binding Accessory Proteins and Cellular Receptors

In herpesviruses, the binding of host cell receptors is performed by either gH/gL itself or a diverse cast of species- or subfamily-specific viral accessory glycoproteins that bind gH/gL. In some cases, gH/gL binds host receptors directly, for example, in the case of EBV and KSHV entry into epithelial cells and, possibly, VZV. However, when gH/gL is not performing that role itself, it recruits viral accessory proteins that can be thought of as “adaptors” for gH/gL. This initial step in the infection process has been best characterized in beta- and gammaherpesviruses.

HCMV, a betaherpesvirus, recognizes several target cell receptors, PDGFRα on fibroblasts and neuropilin-2 on epithelial and endothelial cells, possibly using the ubiquitous receptor tyrosine kinase, TGFβR3, across all host cell types [30,41,42] HCMV engages these receptors using two distinct and mutually exclusive complexes, the trimer (gH/gL/gO) and pentamer (gH/gL/UL128/UL130/UL131) [43,44]. In both complexes, the tropism-determining adaptors are covalently attached to the N terminus of gH/gL [43,44].

Gammaherpesviruses combine adaptor-independent and adaptor-dependent receptor-binding strategies. EBV and KSHV use gH/gL to directly bind the Ephrin A2 receptor on epithelial cells [45,46,47,48,49,50]. EBV also infects B-cells, using viral membrane-anchored accessory glycoprotein gp42 to bind the class II B-cell human leukocyte antigen (HLA II) [45,46,48,49].

The prototypical alphaherpesviruses HSV-1 and HSV-2 use a viral accessory protein gD to bind three known receptors: herpesvirus entry mediator (HVEM), nectin-1, and O-sulphonated heparan sulphate [51,52,53]. Unlike the accessory proteins in beta- and gammaherpesviruses, gD does not form a stable complex with gH/gL but is, instead, thought to bind gH/gL transiently [54,55]. Finally, VZV does not require any accessory viral proteins for viral entry or virus-induced cell–cell fusion (although cell-cell spread of the virus depends on the accessory protein gE) [56]. This suggests that VZV gH/gL binds a receptor directly. Although it remains unclear which host cell proteins serve as the receptors for VZV entry, insulin growth factor receptor and integrins have been implicated [56].

### 3.1. Betaherpesvirinae

**Figure 3:**
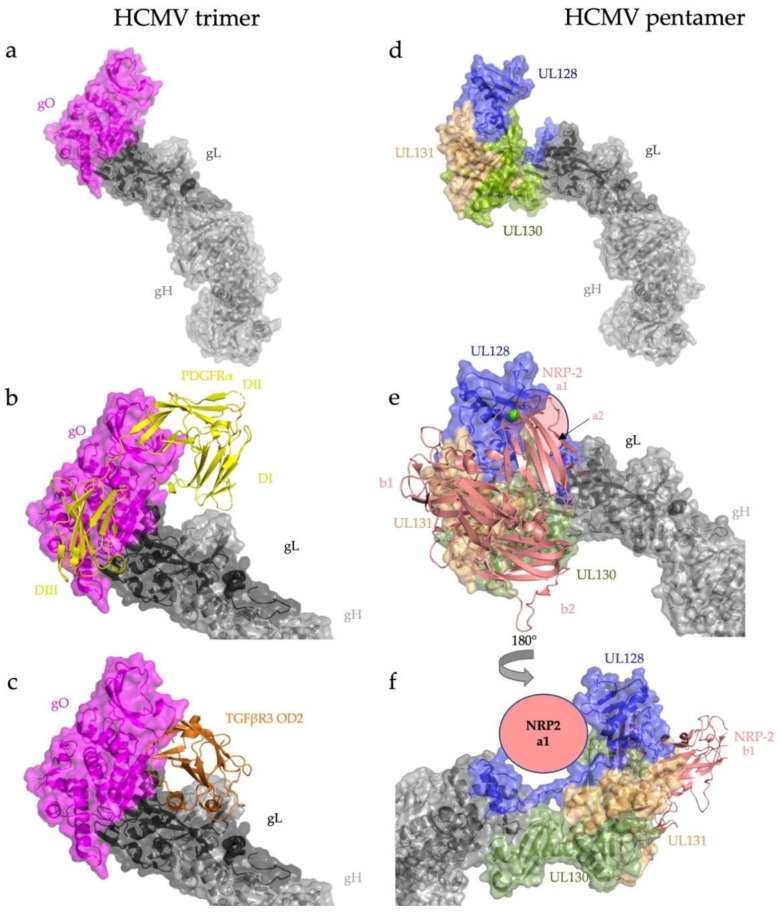
HCMV engages its host cell receptors almost exclusively through the N-terminal adaptors of the trimer and pentamer. (**a**) Overall architecture of the HCMV trimer, gH/gL/gO. The adaptor gO attaches covalently to gL. (**b**) Trimer bound to PDGFRα. PDGFRα domains I-III wrap around gO with minimal contacts with the gH/gL heterodimer itself. (**c**) Trimer bound to TGFβR3. TGFβR3 domain OD2 binds almost exclusively to gO. (**d**) Overall architecture of the HCMV pentamer, gH/gL/UL128/UL130/UL31. (**e**) NRP2 domains a2, b1, and b2 bury a substantial surface across the three components of the pentamer adaptor subcomplex (UL128/130/131), with minimal contact with the gH/gL heterodimer. (**f**) NRP2 domain a1 contacts the C terminus of gL (shown schematically). RCSB PDB IDs: gH/gL/gO/PDGFRα (7LBF) [30], gH/gL/gO/TGFβR3 (7LBG) [30], gH/gL/UL128/UL130/UL31 (5VOB) [38], and gH/gL/UL128/UL130/UL31/NRP2 (7M22) [31].

HCMV has four accessory proteins, gO, UL128, UL130, and UL131, that bind gL [30,38] Both the trimeric (gH/gL/gO) (Figure 3a) and pentameric (gH/gL/UL128/UL130/UL131) (Figure 3d) complexes are covalently linked by a single disulfide bond between the same gL cysteine and the adaptors (gO or UL128), and the amino acid sequence surrounding them is highly conserved across HCMV strains, underscoring their importance to viral replication [30,57].

Receptor binding by both the trimer and pentamer is primarily mediated by the accessory proteins, with minimal contributions from either gH or gL. The ligand-binding domains I, II, and III of PDGFRα, the main host cell receptor for HCMV entry into fibroblasts, wrap around gO, making extensive contacts. There is only one contact point between PDGFRα DI and gH (Figure 3b). The domain O-D2 of TGFβR3 contacts the trimer at three sites, mostly interacting with gO (Figure 3c), and to a lesser extent with gL [30,57].

Binding of the HCMV pentamer to the epithelial and endothelial receptor neuropilin-2 (NRP2) is also mediated by the accessory proteins UL128, UL130, and UL131 [31]. Similar to PDGFRα domains I–III, the NRP2 domains a1, a2, and b2 grip the UL128/UL130/UL131 subcomplex akin to a hand (Figure 3e). Although the majority of the pentamer–NRP2 interaction is mediated by UL128, UL130, and UL131, NRP2 a1 makes a single contact with gL at the gL-UL130 junction (Figure 3f), which is reminiscent of the minimal contributions of gH and gL in receptor recognition by the trimer (Figure 3b,e).

### 3.2. Gammaherpesvirinae

**Figure 4:**
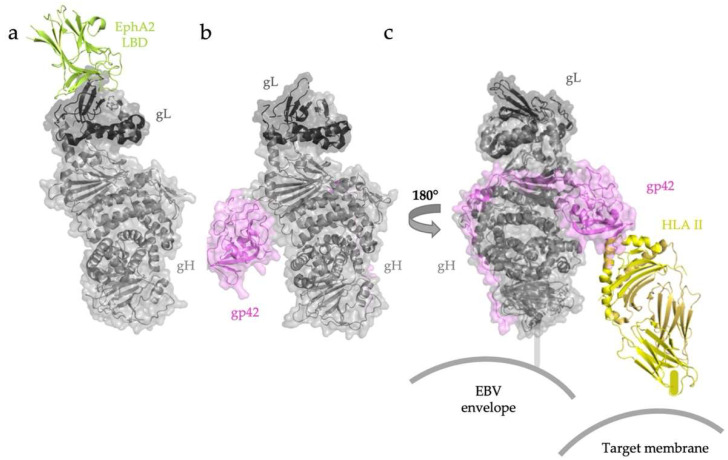
EBV gH/gL recognizes target cell receptors in adaptor-independent and adaptor-dependent ways. (**a**) Both EBV and KSHV (not shown) attach to epithelial cells by binding the EphA2 LBD through extensive contacts with gL. (**b**) Overall architecture of the EBV gH/gL-gp42 complex. (**c**) Interactions with B-cell HLA II are mediated by the globular C-terminal domain of gp42. Once this receptor is engaged, a change in the angle between HLA II and gH/gL has been proposed to trigger fusion. RCSB PDB ID: EBV gH/gL/EphA2 (7CZE) [32]. The structure of EBV gH/gL/gp42/HLA II was assembled from gH/gL/gp42/E1D1 (5T1D) [60] and gp42/HLA II (1KG0) [58].

In members of the gammaherpesvirus subfamily, EBV and KSHV, gH/gL heterodimers directly bind the epithelial cell receptor EphA2 [32,50,58,59] (Figure 4a). In both EBV and KSHV, gL contributes the majority of the binding surface with the ligand binding domain (LBD) of EphA2 [32]. The EphA2-binding regions in EBV and KSHV gH/gL complexes are nearly identical.

EBV entry into human B cells is mediated by an “edge-on” interaction between gH/gL and gp42 bound to HLA II (Figure 4b) [58,60,61]. X-ray crystallography and negative-stain electron microscopy studies have visualized the binding of EBV gp42 to HLA II alone and within the gH/gL/gp42-HLA II complex, respectively. Although the globular C-terminal domain of soluble gp42 binds HLA II with nanomolar affinity, this domain has only weak interactions with the gH/gL heterodimer. The complex is additionally stabilized by extensive interactions between the gp42 N terminus and domains DII–DIV of gH. Three-dimensional reconstruction of negative-stained gH/gL/gp42/HLA II revealed a tripartite architecture to the holocomplex, with gp42-bound HLA II oriented almost parallel to the edge of gH DII (Figure 4c) [59]. This configuration is thought to represent the “closed” conformation of the gH/gL/gp42-HLA II complex, presumably formed at later stages in membrane fusion because it would require the membranes of both EBV and the target B-cell to be in proximity. This conformation may thus be promoting membrane fusion.

### 3.3. Alphaherpesvirinae

gD serves as the receptor-binding protein for HSV-1 and HSV-2. Although no structures have yet been solved of a gD-gH/gL complex, it has been captured and its affinity measured by surface plasmon resonance [62]. Furthermore, antigenic and mutational analyses have suggested that in both HSV-1 and HSV-2, gH domain H1 and gL contribute to the gD-binding surface [29,62,63,64]. An in-depth analysis of gD–gH/gL interactions in HSV can be found in Section 4.3.

By contrast, VZV lacks a gD homolog. Moreover, gB and gH/gL are both necessary and sufficient for cell–cell fusion and formation of the syncytia—multinucleated cells—that are a hallmark of varicella (in skin) and fused neurons and glial cells in zoster (in sensory ganglia) [56,65]. Although gE is required for VZV cell-to-cell spread, it is not required for cell fusion [65]. Therefore, VZV gH/gL itself likely binds a host-cell receptor. Further work is needed to identify a specific host-cell receptor for VZV and characterize its interactions with the virus.

### 3.4. Common Features of gH/gL Interactions with Host Cell Receptors

Across the *Herpesviridae* subfamilies, the N-terminal, membrane-distal module (gL and gH H1/DI) mediates binding to the host-cell receptor or the accessory protein in nearly all cases. In HCMV gH/gL, this region binds gO in the trimer and UL128/UL130/UL131 in the pentamer. In both EBV and KSHV gH/gL, a similarly located region binds EphA2. Finally, as will be discussed in detail in Section 4.3, in HSV gH/gL, this area has been proposed to contain the gD-binding region.

By contrast, HLA/gp42 binds gH/gL “edge-on”, suggesting that the narrow edge of the complex serves a role in receptor recognition. Interestingly, the HCMV pentamer binds a second copy of NRP2 along the side of gH/gL, albeit on the opposite face [31]. While this latter binding event has low affinity, it may be important for either initial binding or conformational rearrangements of the holocomplex into a configuration that can activate gB.

## 4. gH/gL Binding to Antibodies

### 4.1. Betaherpesvirinae

**Figure 5:**
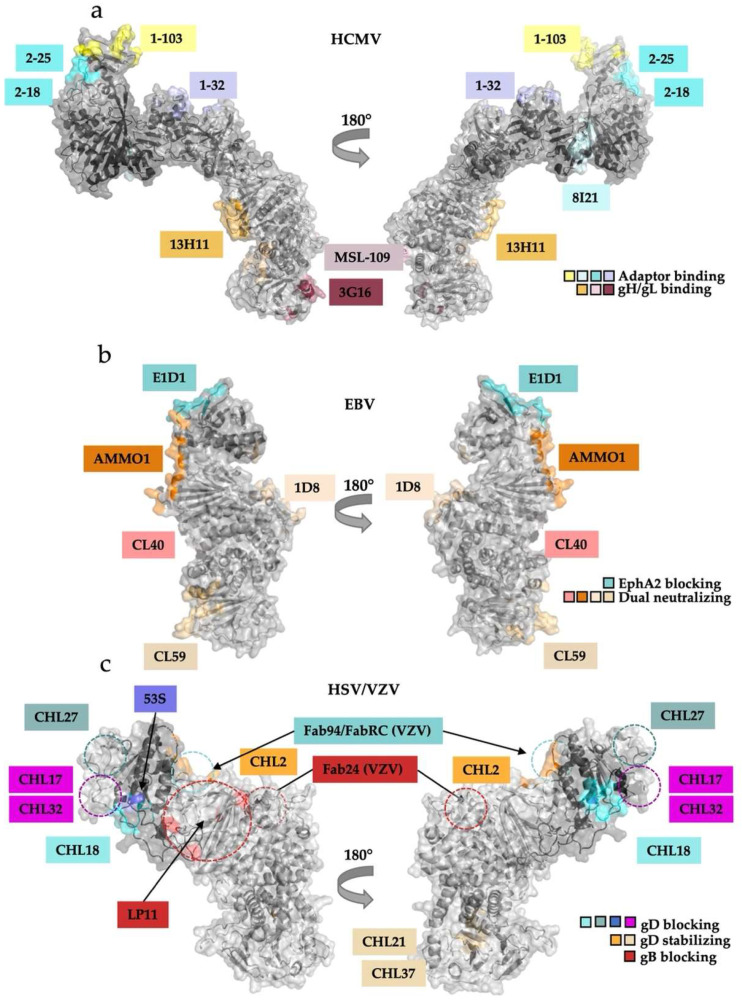
Antibody binding locations on the gH/gL suggest their mechanisms of action. (**a**) Antibodies against HCMV pentamer bind either the gH/gL heterodimer or the adaptor subcomplex. 13H11, MSL-109, and 3G16 neutralize infection of both fibroblasts and epithelial cells whereas the pentamer-binding neutralizing Abs only prevent infection of epithelial or endothelial cells. (**b**) EBV-neutralizing Abs interfere with EphA2 binding but not gp42 or HLA-II binding. E1D1 prevents infection of epithelial cells by sterically interfering with EphA2 engagement by gL. AMMO1, CL40, and CL59 block infection of both epithelial and B-cells without interrupting receptor engagement. (**c**) A battery of antibodies and antibody fragments (Fabs) that bind gH/gL exert different effects on HSV entry. They can be grouped according to their effects: blocking gD binding, stabilizing gD binding, or blocking gB binding. The effects are localized to specific regions of gH/gL. The existing structure of HSV-2 gH/gL is missing the first 29 amino acids of gH after the signal sequence; the approximate locations of the epitopes that map to this region (CHL27, CHL17, CHL32) are indicated schematically with dashed circles. Approximate locations of the LP11 epitope and the epitopes of three Fabs that bind VZV gH/gL are indicated schematically on the HSV-2 gH/gL structure with dashed circles.

In HCMV, the gH/gL complexes are major targets of neutralizing antibodies during natural infection and in immunized mice. Known neutralizing antibodies fall into two classes: those that bind the epitopes within the gH/gL heterodimer itself, even in the context of the pentamer or the trimer, and those that bind the accessory proteins [30,31,66,67]. Neutralizing epitopes within gH have been localized by negative-stain and cryoelectron microscopy to domain IV (3G16), domain III (MSL-109), and the junction of domains II and III within the “kink” of the gH/gL boot (13H11) [30,44,66] (Figure 5a, left). These three epitopes are distal from the receptor-binding regions and thus unlikely to interfere with receptor engagement. Instead, it has been speculated that these antibodies may block the gB–gH/gL interaction required to trigger fusion [30,66]. The neutralizing properties of these three antibodies emphasize the potential role of the membrane-proximal C-terminal half of gH in membrane fusion.

Unsurprisingly, pentamer-specific neutralizing antibodies target the accessory proteins UL128, UL130, and UL131. Twenty known antibodies map to seven distinct sites within UL128-131 subcomplex [66]. Three of these antibodies (1–103, 2–25, 2–18), which bind UL128 and the UL130/131 module, can block interaction with the NRP2 receptor as Fabs (Figure 5a) [31].

The other seventeen antibodies bind in similar regions to the three Fabs mentioned above and, most likely, also neutralize by interfering with receptor binding. As mentioned in Section 3.1, NRP2 a1 binds at the junction of gL and UL128. Fab 1–32 binds essentially the same site and, presumably, blocks NRP2 binding (Figure 3f and Figure 5a, right) [31].

In HCMV, the antibodies targeting the gH/gL complexes appear to neutralize either by blocking receptor binding or interfering with downstream functions, likely, activation of gB. To our knowledge, there are no studies mapping HCMV-neutralizing antibody binding to gO. However, sera of patients infected with HCMV were found to contain both trimer- and pentamer-specific neutralizing antibodies [67]. This clinical study correlates with previous work showing that both trimer and pentamer are required for entry into all cell types [68]. Even though the trimer-specific epitopes have not yet been localized to gO, it is plausible that at least some of the trimer-specific antibodies bind gO rather than gH/gL and block the binding of gO to the fibroblast receptor PDGFRα or to TGFβR3.

### 4.2. Gammaherpesvirinae

Recombinant mouse EBV-neutralizing antibodies that target the gH/gL complexes fall into two distinct categories: epithelial-specific (i.e., those that neutralize EBV entry into epithelial cells but not B cells) and dual-specific (i.e., those that neutralize EBV entry into both epithelial cells and B cells). The epithelial-specific antibody E1D1 [47] binds both the N and C termini of gL at the membrane-distal end of the complex but not gH (Figure 5b) [60]. The epitope of E1D1 overlaps the binding site of EphA2, the epithelial cell receptor [32,60]. However, E1D1 does not block the formation of the gH/gL/gp42 complex or its interactions with the HLA II receptor and can even bind the gH/gL/gp42 complex [69]. Therefore, E1D1 specifically neutralizes the infection of epithelial cells by blocking gH/gL interactions with EphA2.

By contrast, most dual-specific antibodies bind different regions within gH/gL but do not block binding to any known cellular receptors. One of these, CL40, binds at the DII–DIII junction, where the globular C-terminal domain of gp42 is located in the structures of the EBV B-cell entry complex (Figure 4b and Figure 5b). Although CL40 would be expected to interfere with gp42 binding to gH/gL, it can bind both gH/gL and gH/gL/gp42 complexes and does not block binding of the latter to the HLA II receptor [69]. AMMO1, another dual-specific antibody, binds at the junction of gH DII and gL, contacting residues across both proteins, and likewise does not block binding of gH/gL to gp42 [70]. Both CL40 and AMMO1 bind gH/gL in the vicinity of the gp42-binding site, but even though the globular domain of the adaptor protein is normally positioned where it would interfere with AMMO1 and CL40 binding, it can be displaced without disrupting the gH/gL/gp42–HLA II interaction [59,60,69]. This observation highlights the importance of the high affinity interactions between the gp42 N terminus and gH/gL C-terminal domains II, III, and IV.

Finally, CL59, which binds the membrane-proximal DIV, also does not disrupt receptor engagement, which is consistent with its epitope being located far from any known receptor-binding region [69] (Figure 5b). A newly discovered human antibody isolated from EBV-positive patients, 1D8, binds an epitope on the opposite side of gH/gL from the epitopes of AMMO1 and CL40 and blocks virus binding to both epithelial and B-cells [71]. Thus, the dual-specific antibodies appear to neutralize by blocking post-receptor-binding steps, possibly, gB activation. While some may block gB binding to gH/gL others may preclude conformational changes within gH/gL necessary to activate gB.

### 4.3. Alphaherpesvirinae

#### 4.3.1. HSV-1 and HSV-2

A panel of mouse monoclonal antibodies raised against HSV-1 or HSV-2 gH/gL was used to identify regions important for viral entry [72]. Based on their biochemical properties, these antibodies have been sorted into three distinct groups: (1) those that block the gD–gH/gL interaction (CHL27, CHL17/32, CHL18, and 53S), (2) those that stabilize the gD–gH/gL interaction (CHL2, CHL21, CHL37), and (3) one that blocks the gB–gH/gL interaction (LP11) [29,62,64,72] (Figure 5c).

The epitopes of these antibodies have been mapped using a combination of overlapping peptide binding analysis, locations of monoclonal antibody resistance mutations (mar), mutagenesis, and *in-vitro* binding competition assays [62,72,73,74]. Antibodies that block the gD–gH/gL interaction (CHL27, CHL17/32, CHL18, 53S) [62] likely neutralize infection by blocking the gD–gH/gL interaction. Their epitopes cluster at the membrane-distal end of the boot [62], pinpointing the approximate location of the gD-binding site (Figure 5c).

The three antibodies that stabilize the gD–gH/gL interaction (CHL2, CHL21, CHL37) bind two different locations on the gH/gL heterodimer (Figure 5c). CHL2 binds at the membrane-distal end of the boot but on the opposite edge from where the gD-blocking mAbs bind. By contrast, CHL21 and CHL37 bind at the membrane-proximal end of gH, at the junction of H2 and H3. How these antibodies stabilize the gD-gH/gL complex remains unclear. Although none of these three antibodies are neutralizing, they inhibit cell–cell fusion [62].

The HSV-1-neutralizing antibody LP11 blocks gH/gL interactions with gB, and its epitope near the “kink” of the gH/gL boot (Figure 5c) has thus been proposed to overlap the gB-binding site on gH/gL [29]. Interestingly, the binding of LP11, which prevents gH/gL–gB interactions, also abrogates the binding of antibody 53S, which blocks the gD–gH/gL interaction [62]. Since the epitopes of 53S and LP11 do not overlap, LP11 may block binding of 53S through an allosteric mechanism. Further studies are needed to elucidate the inhibitory mechanisms of the anti-HSV antibodies.

#### 4.3.2. VZV

The neutralizing antibodies targeting VZV gH/gL (RC, 94, and 24) bind two adjacent but distinct regions on the membrane-distal edge of the gH/gL boot [37] (Figure 5c). Fabs RC and 94 bind the surface of the N-terminal DI-gL module. Given that the membrane-distal H1/DI in other herpesviruses engages either the accessory proteins or the receptors themselves, these anti-VZV antibodies may neutralize infection by blocking binding of an as-yet unidentified receptor. Fab 24 binds lower down on the gH/gL, closer to the LP11 epitope. Therefore, this antibody could potentially block binding to gB. These hypotheses await further mechanistic analyses to correlate findings between HSV-2 and VZV neutralizing antibodies.

## 5. gB-gH/gL Interactions

While structures of gB in both the prefusion [33,34,75] and the postfusion states [24,25,35,36,76] have clarified the conformational rearrangements in the gB fusogen, how these changes are triggered by gH/gL and even the nature of gH/gL interactions with gB remain unclear.

But whereas in most herpesviruses the gH/gL-gB complex has been thus far elusive, in HCMV, a large proportion of the gH/gL heterodimer that is not bound to gO or UL128/130/131 appears to be stably bound to gB soon after synthesis in the endoplasmic reticulum [77]. Furthermore, cryoelectron tomography of the HCMV glycoproteins on the virion surface has revealed structural features that match a modeled prefusion gB-gH/gL complex [75]. This suggests that, at least in HCMV, gB and gH/gL form a constitutive, possibly autoinhibitory complex that may have to dissociate to trigger the activation of gB. Further work is necessary to determine whether this ternary complex is unique to HCMV and other betaherpesviruses or is conserved across other subfamilies.

**Figure 6:**
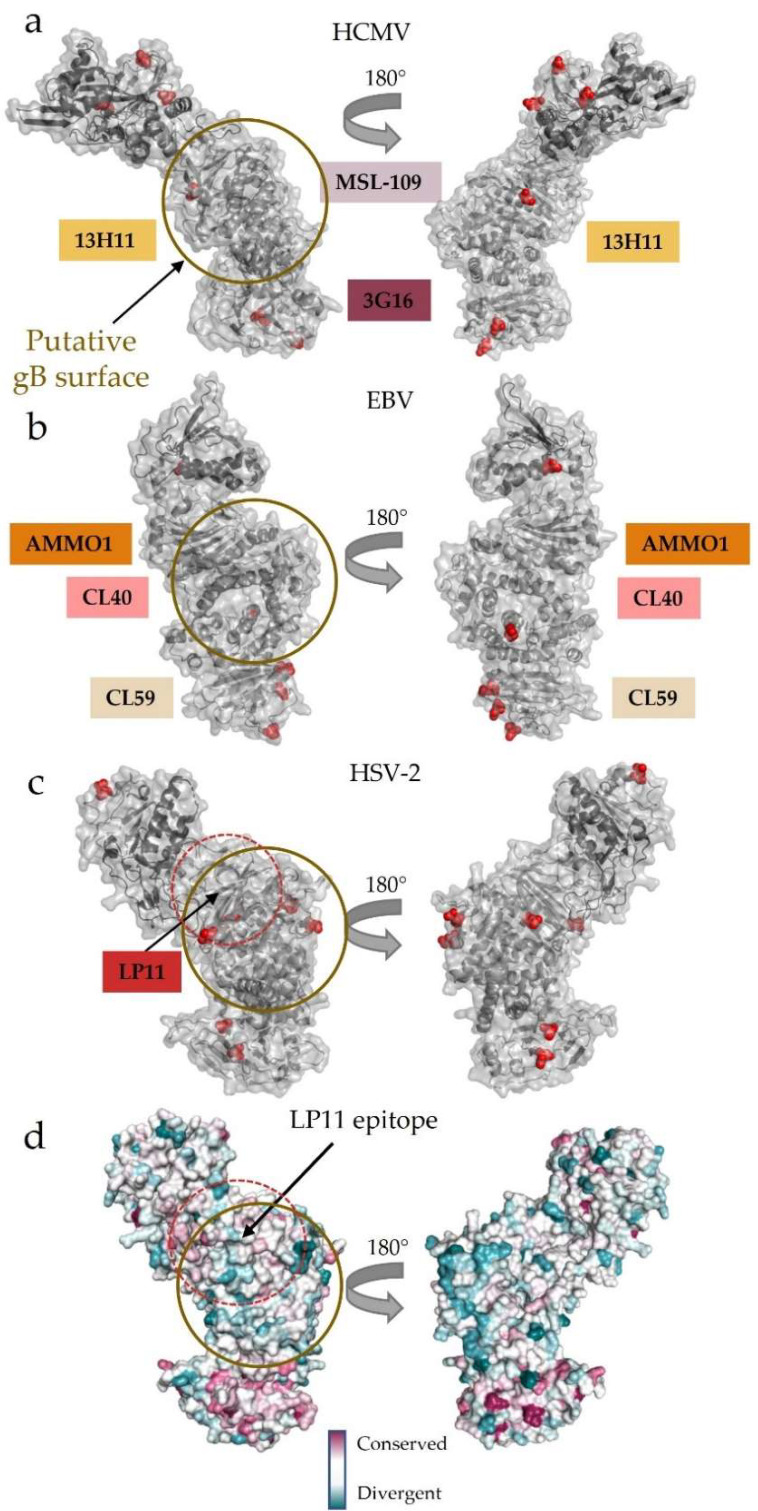
Surface conservation and glycosylation patterns pinpoint the putative gB-binding region. (**a**–**c**) Glycosylation patterns across (**a**) HCMV, (**b**) EBV, and (**c**) HSV-2 gH/gL. The putative gB-binding face, as postulated initially in HSV-2, is remarkably free of glycosylation across the human herpesvirus gH/gL structures currently known. The binding locations of neutralizing antibodies that do not affect host cell receptor binding are indicated along the edge of the heterodimer. These antibodies bind DII, DIII, and DIV in HCMV and EBV gH/gL, while LP11 binds HSV-2 gH/gL at the “kink” between H1 and H2. (**d**) Surface conservation across herpesviruses mapped onto the HSV-2 gH/gL structure. The N terminus, which is used to engage a wide variety of host-cell receptors and receptor adaptors, is more divergent than the C terminus. The wide face of gH/gL, where LP11–an antibody that disrupts gB-gH/gL interactions–binds is more conserved than the opposite face of the complex. All human herpesviruses require gH/gL and gB interactions to trigger fusion.

Interestingly, the wide face where LP11 binds HSV gH/gL lacks glycosylation sites in all gH homologs across the *Herpesviridae* subfamilies (Figure 6a–c). Enrichment of glycosylation sites on a viral glycoprotein surface has been speculated to be used to “shield” exposed region from antibodies [78,79,80,81,82]. Conversely, surfaces involved in protein–protein interactions often conspicuously lack glycosylation [30,83]. Furthermore, this region has not yet been implicated in any other physiological binding events besides neutralizing antibody interactions. These observations point to the non-glycosylated face as a potential gB-binding region, conserved across the three subfamilies of *Herpesviridae* (Figure 6a–c). Indeed, the C-terminal domains of gH/gL are much more conserved across the subfamilies than the N-terminal, receptor-, or adaptor-binding domains that contribute to the broad tropism seen across *Herpesviridae* (Figure 6d). The conservation of the C terminus may reflect the need to bind the equally conserved gB glycoprotein.

Structural analysis of mutations that interfere with binding and activity of the HSV-1-neutralizing LP11 antibody, which also blocks gH/gL binding to gB, localized a putative gB-binding region within HSV-2 gH/gL to one of its flat, wide faces (Figure 5c and Figure 6c) [29]. Since the dual-specific anti-EBV gH/gL antibodies AMMO1 and CL40 do not block receptor binding yet effectively neutralize the infection of both epithelial and B cells, they also likely block the gH/gL–gB interaction necessary to trigger fusion [69,84] (Figure 5b). This explanation is bolstered by antibody studies on the anti-HCMV 13H11, which does not block receptor binding in either the trimer or pentamer but prevents fusion, as described above (Figure 5a) [30,69]. Obtaining the structure of the gH/gL-gB complex is of central importance to understanding the activation step for membrane fusion.

## 6. Concluding Remarks

gH/gL is a unique machine at the center of herpesvirus entry. It takes cues from a wide variety of viral and host cell inputs by either directly engaging target host cell receptors or recruiting a variety of accessory proteins using its N-terminal module. Upon tethering to the host cell, gH/gL then transduces these inputs to activate gB, possibly, by using a region on the wide, unglycosylated face. The distribution of these two essential functions across two distinct regions of one machine most likely underpins the broad tropism of herpesviruses. Given its central functions and conservation across *Herpesviridae*, gH/gL is an attractive target for subunit vaccine or reactive treatment development.

Despite relatively low sequence identities across gH and gL homologs, both the general architecture of and interaction surfaces (putative or established) on the complex are remarkably consistent across the three subfamilies of *Herpesviridae*. Host-cell receptors or accessory proteins bind at the membrane-distal end of the heterodimer and engage poorly conserved gL and the N terminus of gH. By contrast, the putative gB-binding sites are located closer to the membrane, within a more conserved C terminus of gH (Figure 6d) [29,38,39]. This combination of the structural plasticity of the N-terminal module and relative conservation of the C terminal domains gives gH/gL the ability to connect the large cast of accessory glycoproteins with the conserved fusogen.

Although the interactions between gH/gL and its adaptor proteins with host cell receptors have been structurally characterized in beta- and gammaherpesviruses, our knowledge of interactions between gD and gH/gL in alphaherpesviruses remains poor. We also do not yet understand how gH/gL and gB interact, nor the role of these interactions in triggering the fusogenic activity of gB. The architecture of the gB-gH/gL complex, visualized by cryoelectron tomography and pulldown assays, awaits detailed structural analysis. Ultimately, the biggest outstanding mystery surrounding gH/gL is how it transfers the signal from host cell receptors and viral accessory proteins to gB. Shedding light on the mechanism of this process will allow for the development of prophylactic and responsive treatment by targeting an absolutely conserved and essential step of herpesvirus entry.

## Data Availability

Not applicable.
